# Signal Detection on the Battlefield: Priming Self-Protection vs. Revenge-Mindedness Differentially Modulates the Detection of Enemies and Allies

**DOI:** 10.1371/journal.pone.0023929

**Published:** 2011-09-01

**Authors:** D. Vaughn Becker, Chad R. Mortensen, Joshua M. Ackerman, Jenessa R. Shapiro, Uriah S. Anderson, Takao Sasaki, Jon K. Maner, Steven L. Neuberg, Douglas T. Kenrick

**Affiliations:** 1 Department of Technological Entrepreneurship and Innovation Management, Arizona State University, Mesa, Arizona, United States of America; 2 Department of Psychology, Metropolitan State College of Denver, Denver, Colorado, United States of America; 3 Sloan School of Management, Massachusetts Institute of Technology, Cambridge, Massachusetts, United States of America; 4 Department of Psychology, University of California Los Angeles, Los Angeles, California, United States of America; 5 Department of Psychology, Florida State University, Tallahassee, Florida, United States of America; 6 Department of Psychology, Arizona State University, Tempe, Arizona, United States of America; Royal Holloway, University of London, United Kingdom

## Abstract

Detecting signs that someone is a member of a hostile outgroup can depend on very subtle cues. How do ecology-relevant motivational states affect such detections? This research investigated the detection of briefly-presented enemy (versus friend) insignias after participants were primed to be self-protective or revenge-minded. Despite being told to ignore the objectively nondiagnostic cues of ethnicity (Arab vs. Western/European), gender, and facial expressions of the targets, both priming manipulations enhanced biases to see Arab males as enemies. They also reduced the ability to detect ingroup enemies, even when these faces displayed angry expressions. These motivations had very different effects on accuracy, however, with self-protection enhancing overall accuracy and revenge-mindedness reducing it. These methods demonstrate the importance of considering how signal detection tasks that occur in motivationally-charged environments depart from results obtained in conventionally motivationally-inert laboratory settings.

## Introduction

Consider the great challenge confronting soldiers facing a hostile insurgency in a foreign country: They must detect subtle and imperfectly diagnostic signals that may reveal whether an approaching individual is an enemy or a friend, and must do so knowing that making an inaccurate decision—in either direction—could bear a mortal cost to self or others. Motivational factors may further complicate the situation. How might the decisions made by soldiers focused on self-protection differ from those made by soldiers who desire revenge on those responsible for killing a platoon-mate in battle?

Signal detection problems such as this one—with strong functional relevance for the perceiver—differ from those typically employed in signal detection research, which tend to be run in laboratories that are motivationally sanitized and unemotional by design. Although understandable in some respects, this convention is problematic in others. Important decisions often occur in social contexts that engage fundamental motivational systems that, in turn, exert substantial influence over cognition; this motivational modulation of cognition may be especially substantial where the cognitive task is as functionally central as threat detection [1]. In the present research we investigate motivational states related to self-protection and revenge-mindedness, and how these influence the detection of signals that identify individuals as enemies or allies.

One promising approach to understanding such influences involves the coupling of *error management theory*—which proposes that people are cognitively biased to minimize the costs of signal detection errors [2], [3]—with a sociofunctional perspective on motivation and cognition—which highlights the role basic biological and social problems had in shaping the links between decision-making and motivation [4], [5]. This combined approach suggests that decision-making will depend on the goals of the perceiver as well as the perceived social and informational context. For example, people are inclined to over-perceive signs of interpersonal threat when they are feeling fearful, but these perceptions tend to be focused on social targets heuristically associated with the greatest potential to do harm: outgroup males [6], [7], [8]. Applying this same logic to the current research, we expect predictable biases in threat detection that will be differentially modulated by the activation of self-protection or revenge-mindedness.

### Error Management and Enemy Detection Biases

Viewing novel faces entails automatic encoding of communicative signals and social categories [9], [10]. Because these signals and categories may be differentially associated with the inclination and potential to do harm, error management strategies that minimize one's exposure to threats are likely be relatively automatic as well. Three such signals of threat seem especially likely to signal danger. First, angry facial expressions signal an enhanced likelihood of aggression and can be difficult to ignore [11]. The safer error to make is to incorrectly presume that an angry person intends harm than to incorrectly assume the person is friendly; it is less costly to mistakenly avoid a benign individual than to mistakenly approach a dangerous one. Second, because ingroup members facilitate a wide range of functionally critical goals for one another (e.g., [12]) and intergroup interactions have historically been associated with relatively higher levels of danger [13], [14], biases should, and do, exist to generally see ingroup members more positively (e.g., [15]) and outgroup members negatively, especially when threat-related goals are temporarily active [16], [17]. Third, male faces should also be particularly likely to incur such a bias because human males, relative to females, are more likely to hurt others [18], [7].

A number of studies support the often combinatorial role these three factors play in threat detection processes. An emerging literature on intergroup processing suggests that cues to threat enhance processing of outgroup members (from groups stereotypically associated with harm). These cues include angry expressions and masculinity as well as threatening concepts and objects [16], [19], [20]. For example, Correll and colleagues have shown that, when placed in situations that require discriminating between targets that possess or do not possess weapons, people are more likely to “shoot” unarmed Black males than unarmed White males [21]. Even professional police officers exhibit such biases [22]. Outgroup members can themselves also enhance processing of threat-related stimuli. For instance, viewing Black male faces leads perceivers to more rapidly identify images of guns, even when these images are strongly degraded [19], [23]. Such findings fit within an error management framework. However, existing research has tended to presume underlying motivation, or measure it at the level of individual differences [24], but not directly manipulate threat-relevant goals. We might expect that because primed goals can influence automatic evaluations [25], in the context of self-protection or revenge goals, such biases may be further amplified.

### Accuracy

Whereas error management theory generates clear predictions about biases, it is mute regarding accuracy. Recent research reveals, however, that the activation of important goals can enhance the accuracy of cognitive processing. For instance, thinking about survival enhances performance on a memory task [26], and perceivers focused specifically on self-protection more efficiently encode outgroup male faces—they gain additional memory “bang” for their attentional “buck” [6]. We might thus also anticipate perceivers concerned with self-protection to exhibit enhanced accuracy in the detection and use of cues identifying individuals as enemies and friends.

In contrast, a functional perspective does not predict that revenge should increase accuracy. The purpose of revenge is retaliatory and retributive, and this can often be accomplished with broadly biased responses against whole groups rather than focused responses to the specific individual who elicited the desire for revenge. Furthermore, the anger experienced when we desire revenge may actually work in opposition to self-protective motivations, as anger has been shown to suppress fear in mildly dangerous situations [27]. Revenge thus serves as a nice counterpoint to self-protection, because while both may introduce biases that promote ingroup/outgroup discriminations, they should produce quite different effects when it comes to accuracy.

Standard methods of signal detection analysis can be used to estimate both bias and *sensitivity*, or signal discriminability. Importantly, these effects can be independent of one another (i.e., we can become more sensitive to a discrimination but continue to show a response bias as well). In line with the above reasoning, we hypothesized (1) that activating a goal of self-protection should place the cognitive system on alert, increasing sensitivity relative to revenge motivations (the *self-protective vigilance hypothesis*) and (2) that there would also be enhanced anti-male, anti-angry, and anti-outgroup biases in both motivation conditions (the *intergroup bias hypothesis*).

### Overview of the Present Experiment

We asked participants to make decisions concerning the “friend” or “enemy” status of briefly presented target individuals who were identified with perfectly diagnostic enemy/friend insignia but who differed in terms of perfectly nondiagnostic ingroup/outgroup status, facial expression, and sex. Prior to the decision task, participants visualized scenarios designed to activate one of two motivational systems especially relevant to the lives of soldiers in combat—self-protection and revenge-mindedness.

## Materials and Methods

### Ethics Statement

This research was approved by the IRB at Arizona State University, and all participants read and signed statements of informed consent.

### Participants

249 (86 women) students who identified themselves as not being of Arabic or Middle Eastern background participated in exchange for course credit.

### Materials

Stimuli consisted of black and white photographs of faces, sized at 2×1 1/3 in., that varied across the following dimensions: Ethnicity (Arab or Western European, indicated by the presence or absence of Arabic headdress), Sex (Male or Female), and Expression (slightly Angry or Neutral). Eight faces of each of the eight types were constructed, though any individual participant was randomly assigned to see only half of these faces. Expressions were created by digitally manipulating neutrally expressive faces to lower the inner brow, tighten the mouth, and flare the nostrils. A separate group of 24 participants identified these faces as angry with 100% accuracy, and no one reported suspicion that the expressions were not naturally posed.

The task-relevant stimuli were the letters “E” and “F”, constructed from pieces of the face stimuli so the facial photos could serve as post-stimulus masks. The resulting stimuli were approximately 1/3 in. square. We should emphasize here that the signal that was being detected was separate and distinct from the images of the faces. It was an “E” or an “F” that was temporally sandwiched between two displays of the face image, so qualities of the face images, like the headgear on Arabic targets or the greater variability of the hairstyles for the Western targets, should have little effect on the signal's discriminability.

### Procedure

Participants were informed that they were participating in a simulation of circumstances faced by U.S. soldiers in Iraq and given a simple task: identify whether faces are “enemies” or “friends” based on briefly presented insignias. Participants were told that equal numbers of enemies (and friends) were male and female, angry versus neutrally expressive, and Arab versus Western. These characteristics were explicitly descried as non-diagnostic of enemy status, and great efforts were taken to ensure that participants understood this.

Motivational states were activated by having participants listen to one of three narrated, guided visualization passages: (1) self-protection (participants imagined being ambushed while in a hostile foreign city), (2) revenge-mindedness (participants imagined a friend had been ambushed by a foreign combatant, after which the combatant becomes vulnerable to retribution), or (3) a control scenario (participants imagined visiting a foreign marketplace, which featured the same foreign peoples but involved no threat). Participants were asked to try to experience the emotions described in the scenario, because these emotions “should facilitate performance on the subsequent tasks.”

Following this manipulation, participants engaged in three blocks (32 trials each) of a decision task. In each trial, participants were asked to focus on a fixation point (‘+’) appearing in the center of the screen for 1000 ms. A face then appeared (for 500 ms in block one, 200 ms in blocks two and three), followed by the letter ‘E’ or ‘F’ (for 50 ms in block one, 30 ms in block two, and 10 ms in block three), which appeared in four different places within the contours of the faces to prevent participants from focusing on a single location. Finally, the original face reappeared again as a mask, giving participants 1.5 seconds to identify the face as enemy or friend based on the letter that had appeared by pressing the ‘A’ key or the ‘5’ key on the numeric keypad, labeled with an ‘E’ or ‘F’ accordingly. Each face's status as an enemy or friend was counterbalanced, but remained constant across the three blocks for any given participant.

## Results

We analyzed enemy detection as a function of the type of face, using standard signal detection methods [28], which afford separate indices of signal *discriminability* (d′)—how effectively observers distinguished between insignias—and observer *bias* (c)—the overall inclination to label faces as “enemy” or “friend.” These measures were calculated using faces, not participants, as the unit of analysis, to ensure that the normality assumptions of signal detection theory were met [28], and complete means and standard deviations are included in Table 1.

**Table 1:** Full means for the Bias and d-prime measures as a function of target race, target gender, target expression, and participant condition (standard deviations in parentheses).

Bias					
Race	Gender	Expression	Control	Self-Protection	Revenge
Arab	Female	Angry	0.17 (0.14)	0.3 (0.36)	0.07 (0.26)
		Neutral	−0.12 (0.12)	−0.27 (0.37)	−0.18 (0.38)
	Male	Angry	0.22 (0.28)	0.31 (0.16)	0.28 (0.26)
		Neutral	−0.07 (0.41)	0.07 (0.29)	−0.02 (0.25)
European	Female	Angry	0.1 (0.31)	−0.07 (0.25)	−0.12 (0.29)
		Neutral	−0.56 (0.25)	−0.26 (0.2)	−0.53 (0.18)
	Male	Angry	0.27 (0.36)	−0.03 (0.21)	0.01 (0.2)
		Neutral	−0.39 (0.16)	−0.38 (0.16)	−0.46 (0.33)

Detection Sensitivity (d′)					
Race	Gender	Expression	Control	Self-Protection	Revenge
Arab	Female	Angry	0.99 (0.44)	1.13 (0.29)	0.92 (0.63)
		Neutral	0.97 (0.38)	1.25 (0.22)	0.95 (0.59)
	Male	Angry	1.06 (0.43)	1.19 (0.3)	1.06 (0.45)
		Neutral	1.25 (0.57)	1.24 (0.57)	0.96 (0.4)
European	Female	Angry	1.19 (0.28)	1.12 (0.28)	1.2 (0.63)
		Neutral	1.48 (0.34)	1.41 (0.28)	1.2 (0.43)
	Male	Angry	1.29 (0.29)	1.47 (0.43)	1.03 (0.53)
		Neutral	1.11 (0.37)	1.28 (0.48)	1.26 (0.61)

Participant Sex and Block did not moderate effects relevant to our primary hypotheses, so they are collapsed in subsequent analyses. We conducted two 4-way mixed ANOVAs, with motivational condition (control, self-protection, and revenge) as a within-item manipulation and target sex, target ethnicity, and target expression as between-item factors.

### Bias

At the most general level, there was an overall bias to identify faces as “friends” (grand mean = −0.07, *F*_1,56_ = 9.56, p = .003, η^2^_p_ = .145). Moreover, although participants were explicitly told that ethnicity, facial expression, and sex were not diagnostic of enemy status, all three cues elicited significant biases. Relative to their counterparts, angry faces were more likely to be called “enemy”, *F*_1,56_ = 74.8, p<.001, η^2^_p_ = .578, and female faces were more likely to be called “friend”, *F*_1,56_ = 5.56, p = .022, η^2^_p_ = .090, as were Western faces, *F*_1,56_ = 34.3, p<.001, η^2^_p_ = .380.

**Figure 1:**
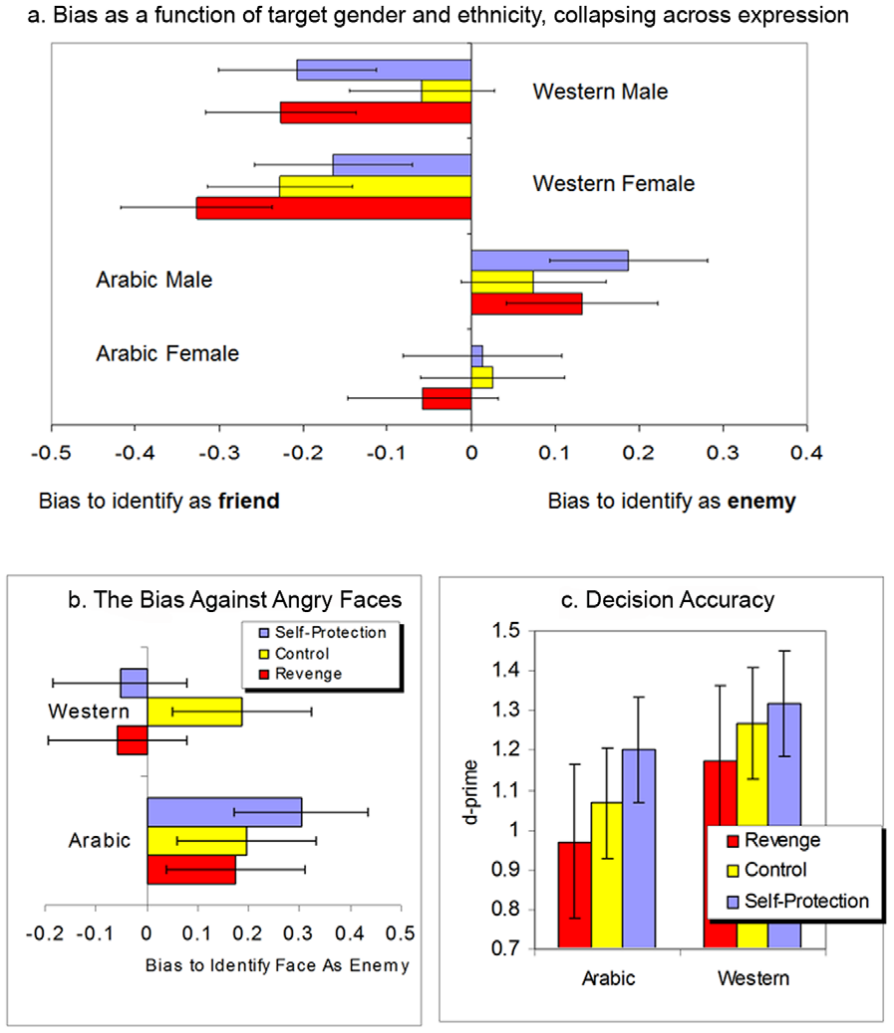
**Figure 1a** shows bias as a function of target gender and ethnicity, collapsed across facial expression. While there is a bias to see Arabic men as “enemies”—and this bias becomes more pronounced in both the self-protection and revenge conditions—there is an even greater bias to call ingroup members “friends”, which entails missing more ingroup enemies. Figure 1b depicts bias for faces showing slight anger (collapsed across target gender), and clearly shows that self-protection and revenge wipe out the bias to call angry ingroup members “enemies”: In the control condition there is a strong bias to call any angry face an enemy, but both self-protection and revenge conditions completely eliminate this bias for ingroup faces. Figure 1c shows participant accuracy in discriminating between enemies and friends.

More importantly, there was an interaction of motivation condition, target race, and target expression, *F*_2,112_ = 4.29, p = .016, η^2^_p_ = .071, supporting the *intergroup bias hypothesis* (see Figure 1b). Whereas control participants were biased to identify angry faces as enemies regardless of whether they were Arabic or Western European (main effect within control: *F*_1,31_ = 7.95, p = .01; no interaction with race), the bias against angry Westerners vanished in both the self-protection and revenge-mindedness conditions (both *F*s<1).

### Signal discriminability

Analyses of d′ within each motivation condition revealed two main effects. First, participants were significantly better at discriminating the insignias of Western faces as opposed to Arabic faces (*F*_1,56_ = 4.72, p = .034, η^2^_p_ = .078). More importantly, supporting the *self-protection vigilance hypothesis*, a main effect of motivation (*F*_2,112_ = 3.95, p = .022, η^2^_p_ = .066) revealed that self-protection concern enhanced accuracy whereas revenge-mindedness decreased it, relative to control (see Figure 1c). In contrast to revenge-mindedness, self-protection motivation enhanced participants' accuracy in detecting enemies while minimizing the false alarms in which they mistakenly labeled friends as enemies, *F*_1,56_ = 4.72, p = .034, η^2^_p_ = .078.

## Discussion

The present experiment revealed motivational modulations of enemy signal detection consistent with an error management approach to heuristic cues to threat. First, in support of our *intergroup bias hypothesis*, there were pronounced biases to label angry faces, outgroup members, and males as “enemies.” Given that it is reasonable to assume that perpetrators of intergroup violence were historically likely to be male and rarely wear the trappings of the ingroup, it makes functional sense that these cues are used to avoid costly errors. Intriguingly, both self-protection and revenge-mindedness essentially eliminated the biases against angry faces of the ingroup, which suggests that coalitional bonds outweigh overt signs of threat (indeed, within the context of observing both ingroup and outgroup males, the angry expressions on Westerners may be interpreted as expressing threat *against the outgroup*, instead of against the self). It is also interesting to note that the “friend” bias for neutrally expressive faces of the ingroup is approximately twice the size of the “enemy” bias for angry faces of the outgroup, which suggests that coalitional biases are aligned more to favor “us” than to disfavor “them”.

In support of the *self-protective vigilance hypothesis*, a self-protection motivation enhanced people's ability to discriminate between the insignias of enemies and friends, while revenge-mindedness motivation gave rise to no such enhancement and trended toward a *decrease* in discrimination performance. In other words, self-protection simultaneously manages two kinds of errors: it minimizes the missing of threats but it also minimizes false alarms calling potential allies “enemies”.

### Conclusion

We found that people were generally inclined to perceive angry, outgroup, and male faces as enemies. A self-protection motivation biased perceivers to view angry outgroup individuals as enemies and ingroup members as friends, but actually increased identification accuracy. Revenge motivation increased the tendency to label outgroup individuals as enemies as well, but it generally decreased the overall accuracy of judgments. We can therefore conclude that self-protection appears to sharpen the senses at the same time that it biases decisions, while revenge-mindedness inclines the perceiver to be biased against outgroup members and in favor of ingroup members, but it does so at the cost of accuracy. We can find little precedent for this effect in the scientific literature, but the idea that revenge dulls decision making is quite consistent with anecdotal accounts of retributive violence in combat situations as well as in counter-insurgency operations.

These findings underscore the utility of investigating how threat-detection can be altered by motivating circumstances. Merely having participants imagine themselves in dangerous situations led to functionally adaptive alterations in signal detection that could have profound consequences under more engaging circumstances in the field. Although these effects may generally reflect traditionally functionally adaptive responses, they may also be quite problematic from the perspective of policy-makers and employers. However, the methods employed here could identify individuals capable of highly accurate decisions under pressure as well as those most prone to dangerous biases, thereby positively informing selection and training for signal detection tasks that occur in emotionally-charged environments. More broadly, the present results suggest that fundamental motivational systems adaptively modulate the detection of social signals, and research programs exploring social perception in this way may lay the groundwork for a more developed model of human information processing in real world circumstances.
